# Significant Pathologic Response Following Neoadjuvant Therapy and Curative Resection in Patients With Rectal Cancer: Surgical and Oncological Outcomes From a Retrospective Cohort Study

**DOI:** 10.1002/cnr2.70041

**Published:** 2024-11-07

**Authors:** Fatemeh Shahabi, Majid Ansari, Khadijeh Najafi Ghobadi, Abolfazl Ghahramani, Amiresmaeil Parandeh, Maryam Saberi‐Karimian, Ala Orafaie, Abbas Abdollahi

**Affiliations:** ^1^ Endoscopic and Minimally Invasive Surgery Research Center Mashhad University of Medical Sciences Mashhad Iran; ^2^ Department of Biostatistics Ilam University of Medical Sciences Ilam Iran

**Keywords:** pathologic complete response, rectal cancer, recurrence, significant pathologic response, survival

## Abstract

**Aim:**

This study evaluated surgical complication rates, recurrence‐free survival, overall survival (OS), and stoma status of patients with rectal cancer after significant pathologic response following neoadjuvant treatment and curative resection. Pathologic complete response (pCR) and near‐pCR patients constitute patients in our study.

**Methods:**

Included was a retrospective cohort study of patients with rectal cancer who were diagnosed between July 2011 and September 2022 and who underwent neoadjuvant therapy and surgical resection.

**Results:**

Of 696 patients with rectal cancer, 149 (21.4%) cases achieved significant pathologic response. During the 64 (70.5) months of follow‐up, recurrence occurred in 16.1% of patients and distant metastases account for the majority of them. Age (*p* = 0.014) and receiving adjuvant chemotherapy (*p* = 0.016) were significantly related to the occurrence of recurrence. The five‐year recurrence‐free survival (RFS) and OS rates were obtained at 83% and 87%, respectively. Although age and surgical technique were significant factors in univariate Cox regression analysis, none of the candidate variables were significant prognostic factors for RFS in the multiple models. The risk of surgical complications remained in these patients. The most frequent complication attributed to infection (20.8%). Despite the 24.8% presence of permanent stoma at primary surgery, more than 50% of our patients lived without stoma at the last follow‐up.

**Conclusion:**

Our recurrence rate was about 16%, and it was related to age and adjuvant chemotherapy. These patients achieved over 80% rates of five‐year RFS and OS. No significant prognostic factors were found on RFS in the multivariable model. As a matter of course, the risk of surgical complications and permanent stoma has still remained in these patients.

## 1 Introduction

Neoadjuvant chemoradiation therapy (CRT) followed by rectal cancer resection has been considered the standard treatment for patients with locally advanced rectal cancer [1, 2, 3]. However, the “watch‐and‐wait” strategy is also a part of the clinical practice guidelines in selected cases [4]. Furthermore, adjuvant chemotherapy, independent of the pathological stage as a part of the latest recommendation for these patients, has been stated [5].

Neoadjuvant CRT can lead to clinical downsizing and downstaging tumors of rectal cancers, with up to 40% of patients having complete tumor sterilization, called clinical complete response [5, 6, 7]. Approximately 15% to 27% of patients will have a pathological complete response (pCR) [5]. pCR after neoadjuvant treatment exhibits reduced local and distant tumor recurrence, increased sphincter preservation, and improved prognosis compared with those without pCR [8, 9, 10, 11]. The appropriate identification of pCR is critical as the chance for prediction of response to neoadjuvant CRT before surgery would allow individualizing treatment [12]. Hence, besides careful patient selection, it is recommended to use sequenced multimodality therapy following a multidisciplinary approach [13].

Recently, it has been shown that in rectal adenocarcinoma patients who undergo neoadjuvant chemoradiation and surgical resection, pCR was associated with excellent long‐term DFS and overall survival (OS), with no evidence of local recurrence over 10 years [14]. Despite excellent survival among pCR rectal cancer patients, as reported by other studies, these cases are still at risk for local or distant recurrence and cancer‐related death [15, 16, 17].

Some of the patients have residual tumor cells or small groups of residual tumor cells in the resected specimen, called near‐pCR [18]. The prognosis of the subgroup with a near‐complete response is controversial [18, 19, 20].

The present study attempts to evaluate oncological and surgical outcomes in patients who achieved significant pathologic response after neoadjuvant therapy followed by surgical resection in the long‐term multicenter follow‐up study. Moreover, the pathologic response status (complete or near response) of our patients was considered as a variable in the evaluation.

## 2 Methods

### 2.1 Patients and Characteristics

**FIGURE 1:**
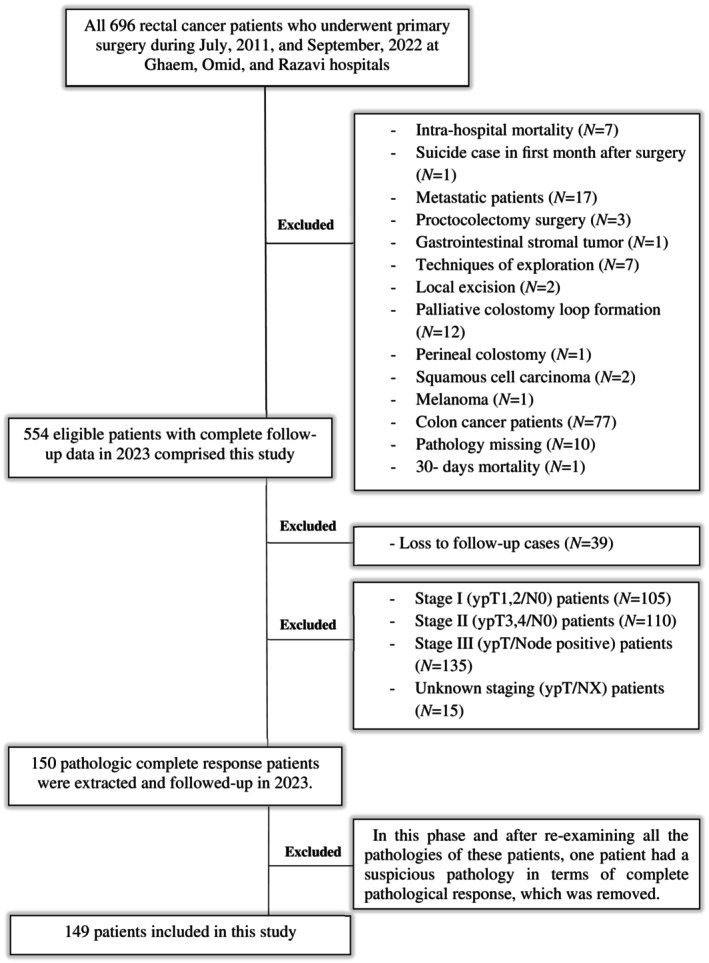
The diagram of patients' recruitment in this study.

No evidence of tumor (ypT0N0) and single cells/rare small groups of cancer cells in the resected specimen were considered pathologic complete response (pCR) and near‐pCR, respectively. These patients were selected from a retrospective cohort study which comprises 696 patients with rectal cancer who were diagnosed between July 2011 and September 2022 and received primary surgical procedures at two universities and one private hospital. The diagram of patients' inclusion and exclusion criteria is illustrated in Figure 1. The ASA indexing score was used to evaluate patients before surgery, and all patients involved in the study had a score of II or III. Neoadjuvant chemoradiation was given to all patients in this study, and surgery was performed 8–12 weeks following the neoadjuvant course. In neoadjuvant treatment, capecitabine 1 g/m^2^ BID accompanied by a total of 28 sessions of radiation therapy with a total radiation dose of 50.4 Gray (Gy) was conducted. Adjuvant chemotherapy (capecitabine and oxaliplatin) was administered for 12 –15 weeks to 64.5% of studied patients who had not received total neoadjuvant therapy. Until the fifth year following curative surgery, the studied patients were monitored using standard rectal cancer surveillance. During the first 2 years after surgery, patients were followed up every 3 months. Afterward, follow‐up appointments were scheduled every 6 months until the fifth year. Our patients receive phone calls every year after these periods. The last follow‐up was scheduled for 2023. Demographic and clinical characteristics of age at diagnosis, gender, surgical techniques, pathologic response status(complete/near), surgery type, surgery duration, and tumor location (the rectum is divided into three parts: the lower rectum, which measures 0 to 5 cm, the middle rectum, which measures 6 to 10 cm, and the upper rectum, which measures 11 to 15 cm), recurrence status (none/yes), recurrence site (only locoregional (LR)/only distant metastasis (DM) or DM + LR), stoma status, and distal resection margin (DRM) status were reported in this study. In addition, postoperative complications of obstruction, anastomosis failure (leakage or stricture), and infectious complications (pelvic collection/abscess/surgical site infection) were reported. The severity of postoperative complications of our patients based on received treatments was reported based on the Clavien‐Dindo classification [21]. The OS and recurrence‐free survival (RFS) known as oncologic outcomes were the primary endpoints, and surgical outcomes were the secondary endpoints of the study. A part of the data of this study was collected from the colorectal cancer registry (No: 4001728), Mashhad University of Medical Sciences, Mashhad, Iran.

### 2.2 Surgery Method

Bowel preparation was done with PEG‐electrolyte lavage solution and oral metronidazole, a day prior to surgery. In addition, preoperative prophylactic antibiotics including cephazolin and metronidazole were used half an hour before surgery. In a modified lithotomy position with a laparoscopic/open approach, after complete abdominal exploration, the inferior mesenteric artery was exposed and highly ligated, and then ligation of the inferior mesenteric vein was done at the inferior border of the pancreas just lateral to the duodenum. Complete mobilization of the splenic flexure colon, left colon, sigmoid, and rectum was done. After complete mobilization, the rectum was cut at enough distal margin and then specimen removal was done through the wound protector from the abdomen (Pfannenstiel incision in laparoscopic surgery). In low/ultralow anterior resection, an anastomosis with a stapler was created between the left colon and distal of the rectum. In addition, coloanal anastomosis was created in the handsewn technique. Finally, for the Hartman and APR procedure, after specimen removal, colostomy was inserted in the left lower quadrant, and for others, diversional ostomy (ileostomy/colostomy) was inserted.

### 2.3 Statistical Analysis

The normal and nonnormal quantitative variables were reported as mean ± standard deviation (SD) and median (interquartile range (IQR)), respectively. Categorical data were expressed in the form of frequency (percentage). The Chi‐square test of independence (if test‐related assumptions were established) or Fisher's exact test (if Chi‐square test assumptions were not established) was rendered to compare categorical variables' frequency distribution between study groups. Two‐sample independent *t*‐tests or Mann–Whitney U tests were used after examining the relevant assumptions to compare the quantitative variables between the study groups. RFS was calculated from the surgical procedure date to the first local recurrence or DM date. OS was evaluated from the surgery to the death date. The endpoint was considered as the last follow‐up for patients without experience of any events (recurrence or death). Cox regression analysis was utilized to evaluate factors' effect on time to event outcome. Factors with *p*‐values ≤ 0.2 remained in the model for multivariable analysis. The figures and analyses were provided using SPSS version 26.0 (Chicago, IL, USA). The significance level was presumed to be 0.05.

## 3 Results

Our study included 149 (21.4%) eligible patients with a mean ± SD age of 55.2 ± 13.2 years. A majority of patients were male (59.1%). Descriptive statistics of the studied patient characteristics were provided in Table 1. Approximately, 74.5% of our patients reached complete pathologic response after surgical procedure and others reached near response. Only 6.7% of patients received total neoadjuvant therapy (TNT) and others received conventional neoadjuvant treatment. In our report, achievement of pathologic complete response or near pathologic complete response was independent of TNT status (TNT received vs. conventional treatment received) in studied patients (*p* = 0.280).

**TABLE 1:** Baseline characteristics of our patients in this study.

Characteristics		
Age at diagnosis, mean ± SD, years		55.2 ± 13.2
Surgery duration, median (IQR), minutes		210 (80)
Gender, *N* (%)	Male	88 (59.1)
	Female	61 (40.9)
Pathologic response status, *N* (%)	Complete	111 (74.5)
	Near	38 (25.5)
Rectal tumor locations, *N* (%)	Lower	89 (59.7)
	Middle	36 (24.2)
	Upper/rectosigmoid	24 (16.1)
Surgical types, *N* (%)	Laparotomy	49 (32.9)
	Laparoscopy	100 (67.1)
Surgical techniques, *N* (%)	Coloanal anastomosis	67 (45)
	Low anterior resection	45 (30.2)
	Abdominoperineal resection/Hartmann's procedure	37 (24.8)
Distal resection margin status, *N* (%)	Normal, DRM > 1 cm	146 (98)
	Short, 0.5 < DRM < 1 cm	3 (2)
Adjuvant chemotherapy status^a^, *N* (%)	*Receipt*	89 (59.7)
	Nonreceipt‐Non‐TNT	49 (32.9)
	Nonreceipt‐TNT	10 (6.7)

Abbreviations: DRM: distal resection margin; TNT: total neoadjuvant therapy.

^a^ 1 (0.7%) case was missing.

**FIGURE 2:**
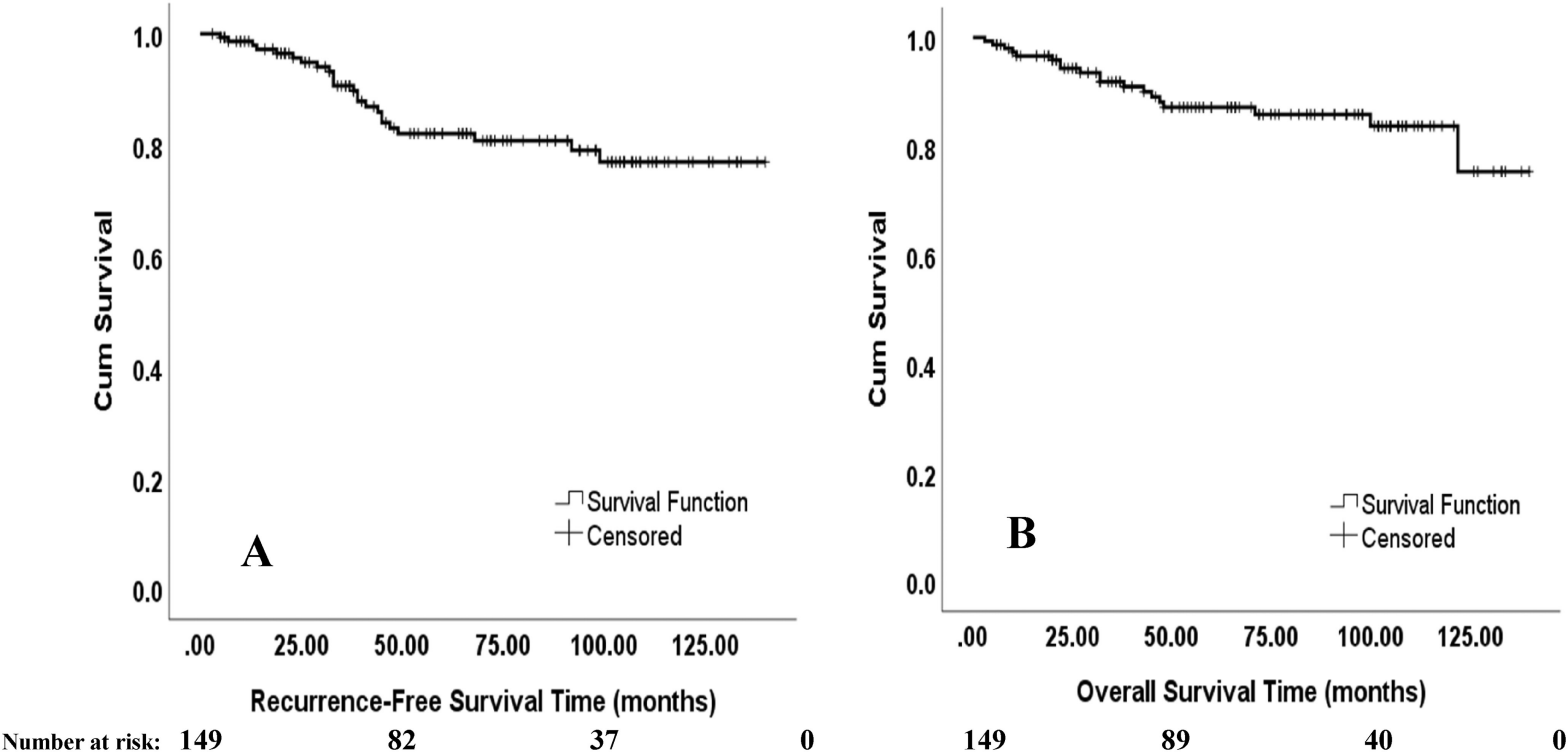
Kaplan–Meier survival curves of all patients included in this study.

Of the studied patients, 24 (16.1%) patients experienced recurrence during follow‐up. Of recurrent patients in this study, the median (IQR) time to recurrence was 35.5 (21.5) months. Table 2 provides the characteristics of recurrent patients with a mean ± SD age of 61.3 ± 12.9 years in our study. 18 (75%), 5 (20.8%), and 1 (4.2%) patients developed DM, LR, and DM + LR, respectively. Moreover, 75% of the recurrent patients related to lower rectal tumor location. More than 50% of recurrent patients did not receive adjuvant chemotherapy. We evaluated the relationship between patients' characteristics, and the development of recurrence is summarized in Table 3. As seen in this table, the mean ± SD age at diagnosis of patients was statistically significantly higher in the recurrent group than in nonrecurrent patients (*p* = 0.014). Furthermore, adjuvant chemotherapy status was not independent of recurrence development (*p* = 0.016). The frequency distribution of pathologic response status (near or complete) was homogenous between recurrent and nonrecurrent groups (*p* = 0.141). Gender, tumor location, surgical types, surgical techniques, and DRM were not related to recurrence development (*p* > 0.05), additionally. The effect of patients ‘characteristics on RFS is demonstrated in Table 4. Although characteristics of the age at diagnosis, pathologic response status (complete/near), surgical techniques, and adjuvant chemotherapy status candidate for entering to multivariable analysis (*p* ≤ 0.2), none of these candidate variables had a statistically significant effect on RFS in the multivariable model (*p* > 0.05) (Table 4). Due to the statistical and clinical correlation between the tumor locations, the surgical technique, and the type of surgery with each other and to prevent collinearity, only one of these three variables entered the regression based on the clinical specialist. Three‐year and five‐year RFS rates ± standard error (SE) were 91% ± 0.02% and 83% ± 0.03, respectively. Kaplan–Meier survival curve of RFS time is illustrated in Figure 2A.

**TABLE 2:** The characteristics of the recurrent patients with pathology results of complete/near response.

Row	Sex	Age	Adjuvant chemotherapy status	Recurrence type	Recurrence site	Tumor location	Dead status	DRM	RFS time (months)
1	M	44	Receipt	Distant	Multiple site	Upper	Alive	Normal	13
2	M	65	Nonreceipt	Distant	Liver	Middle	Dead	Normal	39
3	M	55	Receipt	Distant	Unknown	Lower	Dead	Normal	32
4	M	69	Receipt	Local	Pelvic	Lower	Dead	Normal	32
5	F	43	Nonreceipt	Local	Pelvic	Lower	Dead	Normal	48
6	M	35	Receipt	Distant	Brain	Lower	Dead	Normal	99
7	F	82	Nonreceipt	Distant	Lung	Upper	Dead	Normal	7
8	F	61	Nonreceipt	Distant	Liver	Middle	Alive	Normal	47
9	F	70	Nonreceipt	Local	Pelvic	Lower	Dead	Normal	19
10	M	62	Nonreceipt	Distant	Multiple sites	Middle	Alive	Normal	92
11	M	50	Nonreceipt	Distant	Lung	Lower	Alive	Normal	38
12	M	71	Nonreceipt	Local + distant	Abdomen + lung	Upper	Alive	Normal	29
13	M	82	Nonreceipt	Distant	Lung	Lower	Dead	Normal	45
14	F	54	Nonreceipt	Distant	Liver	Lower	Alive	Short	45
15	M	60	Receipt	Distant	Lung	Lower	Alive	Normal	68
16	M	41	Nonreceipt	Distant	Unknown	Lower	Dead	Normal	5
17	F	71	Nonreceipt	Distant	Lung	Lower	Dead	Normal	23
18	M	86	Nonreceipt	Local	Pelvic	Lower	Dead	Normal	33
19	F	61	Receipt	Distant	Lung	Lower	Dead	Normal	44
20	M	58	Nonreceipt	Distant	Lung/liver	Lower	Dead	Normal	14
21	M	68	Receipt	Distant	Unknown	Lower	Alive	Normal	25
22	M	48	Receipt	Local	Pelvic	Lower	Alive	Normal	39
23	M	63	TNT	Distant	Lung	Lower	Alive	Normal	33
24	M	72	Receipt	Distant	Bone	Lower	Alive	Normal	41

Abbreviations: DRM: distal resection margin; F: female; M: male; RFS: recurrence‐free survival.

**TABLE 3:** Frequency distribution of patients' characteristics between recurrence and non‐recurrence group.

Characteristics		Recurrence	Non‐recurrence	*p*
Age at diagnosis, mean ± SD, years		61.3 ± 12.9	54.1 ± 12.9	0.014^a^
Gender, *N* (%)	Male	16 (66.7)	72 (57.6)	0.408
	Female	8 (33.3)	53 (42.4)	
Pathologic response status, *N* (%)	Complete	15 (62.5)	96 (76.8)	0.141
	Near	9 (37.5)	29 (32.2)	
Rectal tumor locations, *N* (%)	Lower	18 (75)	71 (56.8)	0.228
	Middle	3 (12.5)	33 (26.4)	
	Upper/rectosigmoid	3 (12.5)	21 (16.8)	
Surgical types, *N* (%)	Laparotomy	5 (25)	43 (34.4)	0.369
	Laparoscopy	18 (75)	82 (65.6)	
Surgical techniques, *N* (%)	Coloanal anastomosis	11 (45.8)	56 (44.8)	0.166
	Low anterior resection	4 (16.7)	41 (32.8)	
	Abdominoperineal resection/Hartmann's procedure	9 (37.5)	28 (22.4)	
Distal resection margin status, *N* (%)	Normal, DRM > 1 cm	23 (95.8)	123 (98.4)	0.412
	Short, 0.5 < DRM < 1 cm	1 (4.2)	2 (1.6)	
Adjuvant chemotherapy status^a^, *N* (%)	*Receipt*	9 (37.5)	80 (64.5)	0.016^a^
	Nonreceipt—non‐TNT	14 (58.3)	35 (28.2)	
	Nonreceipt—TNT	1 (4.2)	9 (7.3)	

*Note:* significant *p* ‐values are bold.

Abbreviations: DRM: distal resection margin; TNT: total neoadjuvant therapy.

^a^ significant at *α* = 0.05.

**TABLE 4:** The hazard ratio (95% confidence interval) of the characteristics on time to recurrence using Cox proportional hazard regression model.

Characteristics		Univariate analysis	*p*	Multivariable analysis	*p*
Age at diagnosis		1.05 (1.01.1.09)	0.007	1.03 (0.99.1.07)	0.139
Gender	Male		—		
	Female	0.75 (0.32.1.75)	0.508		
Pathologic response status	Complete	—	—	—	—
	Near	1.66 (0.72.3.79)	0.231	1.38 (0.59.3.26)	0.458
Surgical Techniques	Coloanal anastomosis	—	—	—	—
	Low anterior resection	0.74 (0.23.2.33)	0.607	0.64 (0.20.2.07)	0.462
	Abdominoperineal resection/Hartmann's procedure	2.76 (1.12.6.81)	0.027	2.10 (0.74.5.94)	0.162
Distal resection margin status	Normal, DRM > 1 cm	—	—		
	Short, 0.5 < DRM < 1 cm	1.45 (0.19.10.7)	0.718		
Adjuvant chemotherapy status	Nonreceipt—non‐TNT	—	—	—	—
	Receipt	0.44 (0.19.1.01)	0.054	0.54 (0.22.1.32)	0.176
	Nonreceipt—TNT	1.45 (0.18.12.0)	0.729	0.86 (0.10.7.41)	0.893

*Note:* significant *p* ‐values are bold.

Abbreviations: DRM: distal resection margin; TNT: total neoadjuvant therapy.

* significant at *α* = 0.05.

The median (IQR) survival time of all 149 pCR/near pCR patients was 64 (70.5) months. The mortality rate in our subjects was 12.8%. Three‐year and five‐year OS rates ± SE were 92% ± 0.02% and 87% ± 0.03, respectively. OS Kaplan–Meier curve is shown in Figure 2B.

Intraoperative complications of bleeding and internal organ injuries were reported in 5 (3.4%) and 2 (1.3%) of patients, respectively, and 0.7% (1 case) and 20.8% (31 cases) of postoperative morbidity rates belong to obstruction and infectious complications. Moreover, anastomosis failure happened for 17% (19 subjects) of patients who underwent surgical procedures with the anastomosis technique.

87.9% (51) of all complications were postoperative. According to Clavien‐Dindo classifications, postoperative complications were classified as follows: grade I (4%, 7.8%), grade II (1%, 2%), grade IIIa (4%, 7.8%), and grade IIIb (42%, 82.3%)

**FIGURE 3:**
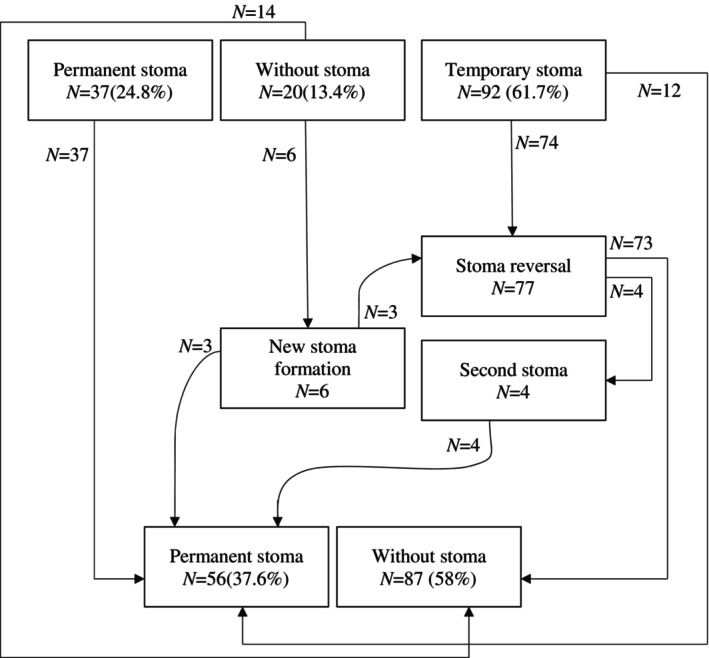
Stoma status in our patients that included in this study.

Stoma‐related events are illustrated in Figure 3. This figure described the stoma life in pCR/near pCR rectal cancer patients. However, 37 (24.8%) patients underwent surgery with permanent stoma at primary surgery, and more than 50% of our patients lived without stoma at the last follow‐up. More than 80% of patients with a temporary stoma in primary surgery had a chance of stoma reversal and 9.4% of patients never experienced a stoma in their life until the last follow‐up. The permanent stoma rate in patients with temporary or without stoma in primary surgery was 22.3%.

## 4 Discussion

The present study has demonstrated that 21.4% of the patients with rectal cancer achieved significant pathologic response (pCR and near‐pCR) followed by neoadjuvant treatment and curative resection. Similar results have been reported in other literature. To illustrate, peng et al. demonstrated that 26.6% of rectal cancer patients achieved pCR [22]. Kim et al. also reported that 23.7% of rectal cancer patients who have undergone surgery after neoadjuvant chemoradiation therapy (nCRT) achieved pCR [23]. Similarly, Zeng et al. shown 23.2% of rectal cancer patients with pCR after nCRT followed by total mesorectal excision [24]. Moreover, in a retrospective study by Tan et al. on 6555 patients, 20.5% of the cases achieved pCR [25]. Our significant pathologic response rate was similar to the aforementioned studies, nevertheless, these studies were not considered near‐pCR. In the Tranchart et al. study, 21.6% of significant pathologic response rate (complete (ypT0) or major (very few viable tumor cells)) was reported in rectal cancer patients after neoadjuvant radiotherapy before resection [26]. The difference between our study and this study was the specimen examination time. Our significant pathologic response belonged to after neoadjuvant treatment and curative resection.

Clinically undetectable residues of tumor or pathologic lymph nodes can remain in the mesorectum after nCRT for rectal cancer which is called a near‐complete response. Despite not meeting all the criteria of clinical complete response, significant tumor downsizing has typically occurred in patients with a near‐complete response [27]. Swellengrebel et al. demonstrated that rectal cancer patients with pCR after nCRT had excellent outcomes. At the same time, an unexpectedly poor prognosis was observed in the subgroup with a near‐complete response [18]. However, we did not observe a significant correlation between the presence of single cells/rare small groups of cancer cells in the resected specimen (near‐complete response) and tumor recurrence. The pivotal difference between our study and the studies mentioned earlier was the definition of near‐pCR. The near‐pCR was assessed after neoadjuvant therapy in those studies, whereas in our study, it was evaluated after neoadjuvant therapy followed by resection. In addition, there is a lack of data on near or complete response pathologic effects on recurrence after neoadjuvant therapy followed by resection in the literature. Hence, it had been questioned how to deal with near‐pCR patients who underwent resection after nCRT. Thus, the question arises whether a near‐pCR after nCRT and surgery translates into pCR. More studies are needed to answer these.

Several investigations have reported that the postoperative pathological stage after nCRT was strongly correlated with prognosis for locally advanced rectal cancer patients. Quah et al. indicated a significant association of postoperative complete or near‐complete pathologic response with DFS, and OS of the patients [28]. Park et al. also revealed that in patients with locally advanced rectal cancer, 5‐year OS and 5‐year RFS for those with ypT0N0 stage were 93.4% and 90.5%, respectively [29]. Furthermore, Sinukumar et al. reported a 3‐year OS of about 94.6% and a 3‐year DFS of about 88.5% [30]. Capirci et al. also reported overall, 5‐year rates of OS and DFS of about 90% and 85%, respectively [31]. In the current study, three‐year RFS and five‐year RFS rates were 91% and 83%, respectively. In addition, the three‐year and five‐year OS rates were 92% and 87%, respectively.

There is controversy about the use of adjuvant chemotherapy for patients with rectal cancer who exhibit pCR. Several studies have been conducted to determine whether the administration of adjuvant chemotherapy improves survival among pCR patients. For instance, the Dutch PROCTOR‐SCRIPT trial on 437 patients with locally advanced rectal cancer reported no difference in OS and a statistically nonsignificant benefit in DFS and the rate of recurrence in the adjuvant chemotherapy group compared with postoperative observation alone [32]. Similarly, Gamaleldin et al. compared pCR patients who received adjuvant chemotherapy with the patients who underwent observation and found no difference in oncologic outcomes among them [33]. On the contrary, Turner et al. demonstrated that in patients with locally advanced rectal cancer with pCR following nCRT, adjuvant chemotherapy was associated with an improved unadjusted 5‐year OS compared to those who did not receive it [16]. In another study, Polanco et al. conducted a study on 2764 locally advanced rectal cancer patients who achieved pCR after nCRT and resection and found that the adjuvant chemotherapy cohort had improved OS compared with the patients who did not receive it [5]. In 2023, Lai et al. studied the impact of adjuvant chemotherapy on 2221 locally advanced rectal cancer who achieved pCR after nCRT followed by total mesorectal excision. They found that OS at 5, 10, and 14 years was significantly longer in patients who received adjuvant chemotherapy [17]. In the present study, we found that adjuvant chemotherapy had a significant correlation with tumor recurrence. More than 50% of our recurrent patients had not received adjuvant chemotherapy and only 4% of them had received a TNT regimen. Although this parameter could not show a significant effect on recurrence in multivariable Cox regression analysis, it was a significant prognostic factor for RFS in the univariate model.

Petrelli et al. in a systematic review of randomized trials demonstrated a residual risk of local recurrence (2.8%) and distant metastases (9%) in pCR patients with rectal cancer who underwent neoadjuvant therapy followed by radical surgery. This systematic review included 22 trials involving 10 050 patients [34]. In the present study with a median (IQR) of 64 (70.5) months of follow‐up, 3.3% of patients developed local recurrence, 12.1% of the cases showed distant metastases, and 0.7% developed both kinds of recurrences. Despite our study which a near‐complete response was also considered, our results were almost consistent with this large systematic review.

Several studies have demonstrated the postoperative complications in pCR patients. As reported by Wolf et al. the 30‐day complication rate for patients with pCR was significantly lower than non‐pCR patients, and pCR is associated with improved postoperative outcomes [1]. Reduced rates of complications in pCR patients might happen because nonresponsive tumors are more challenging to resect, and so these are more prone to technical complications, such as organ space SSI or intraoperative bleeding [1]. However, Duldulao et al. and Landi et al. both revealed that there was no significant difference in the frequency of total surgical complications between pCR and non‐pCR patients [35, 36]. Therefore, pCR cases are not exempt from intra‐ or postoperative complications. Kim et al. reported that 9.6% of pCR patients with postoperative wound complications. In addition, anastomosis leakage was observed in 2.6% of patients and stenosis in 0.9% of the cases; intestinal obstruction was also found in 2.6% of patients [37]. Horisberger et al. also found that the incidences of anastomotic leak and SSI were significant in pCR patients [38]. In the present study, the highest complication rates were for postoperative infectious complications (20.8%) and anastomosis failure (17%), while obstruction had the lowest rate (0.7%) of incidence.

Patients who achieved clinical complete response after nCRT may be recommended to have local excision or a watch‐and‐wait treatment strategy to avoid radical surgery complications and improve quality of life without compromising the oncological outcome [39, 40, 41]. However, in watch‐and‐wait patients, worse survival and a higher rate of DM than pCR cases are notable [42]. To illustrate, Smith et al. reported the five‐year OS and DFS rates of 73% and 75% in the watch‐and‐wait group, while these rates for the pCR group (they underwent total mesorectal excision and had no residual disease on pathology (ypT0N0)) were 94% and 92%, respectively. Furthermore, 8% of the watch‐and‐wait patients developed distant metastases, while 4% of pCR patients experienced this outcome [43]. More recently, Rega et al. also found local recurrence and distant metastases in 15.3% and 12.8% of the watch‐and‐wait patients, respectively. Through a retrograde insight into patients who achieved pCR or near‐pCR, it can be said with a high probability that these patients were probably candidates for the watch‐and‐wait approach before surgery. The survival and recurrence rates in the present study were in line with the results of other similar studies on pCR patients. Our results showed that pCR patients experienced intra‐ and postoperative complications. Moreover, considering abdominoperineal patients, 37.6% of the patients in our study live with a permanent stoma at the last follow‐up session, which of course affects their quality of life. Therefore, in clinical complete response patients, in case of good surveillance and accurate and regular follow‐up, if appropriate treatment is performed at the right time, watch and wait might be considered as an alternative treatment strategy.

The study was limited by its retrospective nature. The strength of our data was the accurate and long‐term follow‐up of the patients. Another strength of this study was the examination of oncological and surgical outcomes together, considering the effect of pathological response status (near or complete response). Furthermore, in this study, the stoma status of the patients had been described in detail. However, this study suggests using a larger sample size which may improve the accuracy of the results.

Based on the literature, patients with pCR have superior postoperative outcomes compared to patients without pCR. However, although pCR is associated with very good long‐term survival, in patients with rectal cancer who achieved pCR/near pCR after neoadjuvant therapy and tumor resection, surgical complications are still visible. In addition, despite the tumor resection, local recurrence and particularly DM are possible in these patients. Therefore, even in these patients, the necessary attention should be paid to postoperative care and regular follow‐up.

## 5 Conclusion

Our findings have shown that about 20% of our patients with rectal cancer achieved pCR/near pCR. Of these, 16.1% developed at least one type of recurrence. Age at diagnosis and receiving adjuvant chemotherapy were significantly related to the occurrence of recurrence in our patients. However, no significant factor affected the RFS in a multivariable time‐to‐recurrence analysis. The three‐ and five‐year RFS rates were 91% and 83%, respectively. In addition, we did not find any significant differences between the prognoses of near‐pCR and pCR groups. Despite the achievement of acceptable survival outcomes in near‐pCR/pCR patients, regular follow‐up based on standard surveillance for rectal cancer remains necessary as the risk of recurrence and even late recurrence still exists in these patients. As well as the risk of surgical complications and permanent stoma has still remained in these patients.

## Author Contributions

**Fatemeh Shahabi:** investigation, methodology, data curation, formal analysis. **Majid Ansari:** supervision. **Khadijeh Najafi Ghobadi:** methodology, formal analysis, investigation. **Abolfazl Ghahramani:** writing – review and editing. **Amiresmaeil Parandeh:** writing – review and editing. **Maryam Saberi‐Karimian:** investigation. **Ala Orafaie:** writing – original draft, writing – review and editing. **Abbas Abdollahi:** supervision, project administration.

## Ethics Statement

The study was done under the consideration of the Mashhad University of Medical Sciences Ethical Committee (code: 4002024). In addition, the Mashhad University of Medical Sciences Ethical Committee has waived the need for informed consent, as this study was a retrospective work.

## Conflicts of Interest

The authors declare no conflicts of interest.

## Data Availability

The datasets used and/or analyzed during the current study are available from the corresponding author upon reasonable request.
